# Long‐term outcome of autogenously transplanted maxillary canines

**DOI:** 10.1002/cre2.159

**Published:** 2019-01-17

**Authors:** Koenraad Grisar, Margaux Nys, Vincent The, Luc Vrielinck, Serge Schepers, Reinhilde Jacobs, Constantinus Politis

**Affiliations:** ^1^ OMFS IMPATH Research Group, Department of Imaging & Pathology, Faculty of Medicine, University of Leuven and Department of Oral & Maxillofacial Surgery University Hospitals Leuven Leuven Belgium; ^2^ Department of Oral and Maxillofacial Surgery St. John's Hospital Genk Belgium; ^3^ Department of Dental Medicine Karolinska Institutet Huddinge Sweden

**Keywords:** autotransplantation, canine, impaction, maxillary, outcome

## Abstract

The aim of this study was to determine the long‐term outcome of autotransplanted maxillary canines and to investigate the influencing parameters.

Seventy‐one patients (84 transplanted canines) volunteered to participate in this study. The mean follow‐up time was 21 years. In case of tooth survival and when patients were found willing for recall, teeth were investigated clinically and radiographically. Transplanted teeth were compared to the contralateral canine and scored with an aesthetic and radiographic index.

The survival rate was 67.9%, considering that 27 transplanted teeth were lost before examination. The mean survival time was 15.8 years.

Maxillary canine autotransplantation may have a successful outcome up to 21 years after transplantation requiring minimal patient compliance and low financial costs. The survival rate can be considered favorable realizing that autotransplantation is a treatment option in a selected group of cases.

## 1 INTRODUCTION

Permanent maxillary canines are essential considering aesthetics, and lip support (Fagade, Gillbe, & Wastell, 1988; Patel, Fanshawe, Bister, & Cobourne, 2011). However, apart from the wisdom tooth, upper canines are the most frequently impacted teeth (incidence 0.9 to 2.2 percent) (McSherry, 1998). Impaction of the permanent maxillary canine occurs two times more often in females (McSherry, 1998). Eight to ten percent of the cases are bilateral (Bishara, 1992).

Canine impaction has been reported to increase orthodontic treatment time, with complicated orthodontic treatment mechanics and increased treatment costs (Barlow, Moore, Sherriff, Ireland, & Sandy, 2009; Zuccati, Ghobadlu, Nieri, & Clauser, 2006).

The traditional treatment options for impacted canines are interceptive removal of the decidiuous canine, surgical exposure with or without orthodontic traction to align the malpositioned tooth, no treatment, autotransplantation of the permanent canine or removal of the permanent canine and prosthetic or restorative treatment.

When surgical exposure and subsequent orthodontic realignment are difficult or impossible due to unfavorable impaction position of a impacted maxillary canine or the patient refuses prolonged orthodontic treatment, autotransplantation is a valuable alternative. Autogenous tooth transplantation can be defined as the surgical movement of a tooth from one position in the mouth to another in the same individual. (Moss, 1968) Few long‐term follow‐up studies have been published in literature (Grisar, Chaabouni, Romero, Vandendriessche, & Jacobs, 2018). The present study aimed to determine the long‐term outcome and survival of autotransplanted canines.

## 2 MATERIAL AND METHODS

### 2.1 Subjects

In 71 patients, 84 teeth, maxillary canine transplantation had been performed. All these procedures were performed between 1995 and 2002. Equal gender distribution was found (33 male (41 teeth) and 38 female (43 teeth) (Table 1). At the time of transplantation the mean age was 20.7 years (range 10.9–46.3 years), and the mean follow‐up period was 21 years (range 19.9–23.9 years). The same surgeon performed all transplantations (CP), following the same protocol. All transplanted teeth reported here were maxillary impacted canines. Pre‐ and perioperative parameters were retrieved out of the medical files (Table 2). By observation of previous radiographs (intra‐oral and panoramic), the stage of root development at time of transplantation was evaluated with Moorrees et al.’s classification. (Moorrees, Fanning, & Hunt, 1963)

**Table 1:** Number of patients, number of transplanted teeth, and age at time of transplantation subdivided by gender

	**N**	**Number of transplanted teeth**	**Age at time of transplantation, mean (SD)**
**Male**	38	46	21.5 (+/− 9.9)
**Female**	33	38	19.9 (+/− 9.5)
**Total**	71	84	20.7 (+/− 9.7)

**Table 2:** Pre‐ and postoperative parameters which could influence the outcome of transplantation

**Preoperative parameter**	**Total (n)**	**Survival (n (%))**	**Failure (n (%))**	**p‐value**
Position of the canine				
Palatal	82	57 (69.5%)	25	0.21
Labial	2	0 (	2	
Sufficient space for transplantation				
Yes	79	53 (67.1%)	26	0.56
No*	5	4	1	
Stage of root development				
1/2–3/4	3	3	0	
>3/4	23	14	9	
Complete	58	40	18	
Condition of apex				
Open	25	19	6	0.31
Closed	59	38	21	
Apical anomaly				
Curved apex	22	15	7	0.97
No curved apex	62	42	20	
Baseline ankylosis of the transplanted tooth				
Yes	19	7	12	<0.005
No	65	50 (76.9%)	15	
Damage of the periodontal ligament				
Yes	15	5	10	<0.005
No	69	52 (75.4%)	17	
Fixation				
Orthodontic wire	65	46	19	
Trauma splint	18	10	8	
No fixation	1	1	0	

All patients were contacted by telephone and survival of the transplanted canine was checked for. In case the transplanted canine was still in situ, patients were invited for a recall visits to the department for further clinical and additional radio graphical analysis. Out of the 47 patients (57 surviving autotransplanted maxillary canines) who were eligible for a recall visit, 23 patients (27 surviving autotransplanted maxillary canines) decided to participate in the presented study. Clinical and radio graphical examination of these 27 autotransplanted maxillary was performed by the same examiner. This involved evaluation of the transplanted canine and the contralateral canine using aesthetic and radiographic indexes as described by Grisar et al (Grisar et al., 2018; Grisar et al., 2018). In case of bilateral autotransplantation both teeth were evaluated and compared with the contralateral canine. In case of absence of the contralateral tooth, only the transplanted tooth was evaluated.

The mobility of the transplanted tooth was tested by means of the Periotest (Medizintechnik Gulden, Modautal, Germany). Periotest measurements were taken and interpreted according to the manufacturer's instructions. Negative Periotest values indicate lower mobility, pointing toward ankyloses (Gonnissen et al., 2010).

The 24 patients (27 failed autotransplanted maxillary canines) that reported a failure of the transplanted canine at the telephone contact were further questioned concerning the timing of failure and the current treatment or treatment plan (no plan, resin retained bridge, prosthesis, dental implant with or without bone augmentation procedure).

The study protocol was approved by the Ethics Committee of our Hospital (s number: s53225).

### 2.2 Surgical procedure

**Figure 1:**
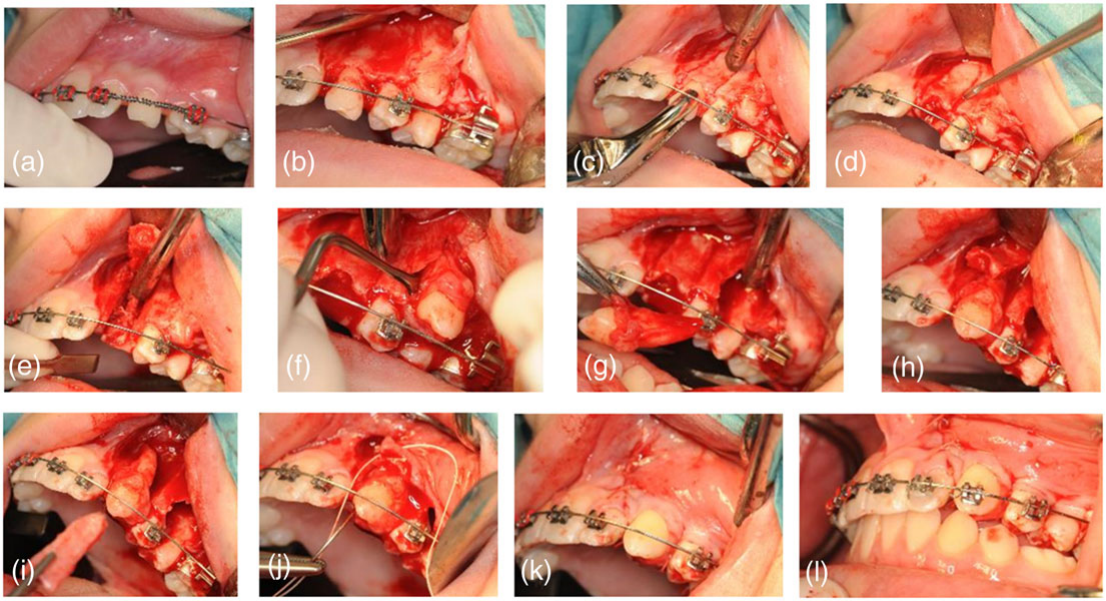
Transplantation of an ectopic maxillary canine. A, Vestibular location. B, trapezoidal incision. C‐D, Osteotomy with a fine surgical drill and chisels. E, Preparation of the recipient socket with chisels. F‐G, Removal of the graft with careful handling of the periodontal ligament. H‐K, Positioning of the donor tooth into the recipient socket and suturing of the trapezoidal flap. L, Fixation in the orthodontic arch with a bracket and orthodontic wire in infraocclusive position

The same surgeon performed all transplantations (CP), following the same protocol. This protocol, including the surgical technique and criteria for endodontic treatment, has been described in the previous study of Gonissen et al (Figure 1). (Gonnissen et al., 2010) Prior to carrying out the actual surgery a radiographic presurgical analysis was carried out identifying the specific location and donor site characteristics.

### 2.3 Clinical and radiographic examination

All transplanted teeth that were still in place were eligible for recall and further evaluation. Each patient signed a written informed consent form approved by the St. John's Hospital Ethics Committee (B371201733373). Clinical evaluation was performed according to the protocol described in the publication by Gonissen et al. (Gonnissen et al., 2010) Tooth vitality, tooth mobility (Perio‐test), gingival inflammation, pocket status and aesthetic outcome were scored. Aesthetic outcome was assessed with the maxillary canine aesthetic index (MCAI) as described by Grisar et al. (Grisar, Claeys, et al., 2018).

Intraoral radiographs (Sirona, 70 kV, 0.06 s, 7 mA) and conebeam computerized tomography (CBCT) images of all transplanted teeth were taken. At the OMFS department of the St. John's Hospital, the cone‐beam scanner Galileos (Sirona, 85 kV, 7 mA, 14 s, 15 cm^3^) is used. Radiologic examination allowed evaluation of root resorption, periodontal ligament and lamina dura formation, ankylosis, alveolar bone loss, and apical inflammation. Radiographical outcome of the transplanted canine was assessed with the autotransplanted maxillary canine radiographical index (AMCRI) as described by Grisar et al. (Grisar, Vanpoecke, et al., 2018).

### 2.4 Statistical analysis of the results

The ratio between failed and succeeded canines was first compared between different groups by means of a generalized linear model. Subsequently, survival analysis was performed by means of Kaplan–Meier graphs and survival regression for censored normally distributed data.

## 3 RESULTS

### 3.1 Clinical investigation

Twenty seven transplanted maxillary canines were examined. Almost half of the teeth (17 teeth) showed negative Periotest values. Periotest values higher than the normal values were found with 2 teeth. The remaining 8 teeth had normal Periotest values. 2 transplanted teeth showed grade 2 tooth mobility. None of the contralateral canines showed altered mobility.

**Figure 2:**
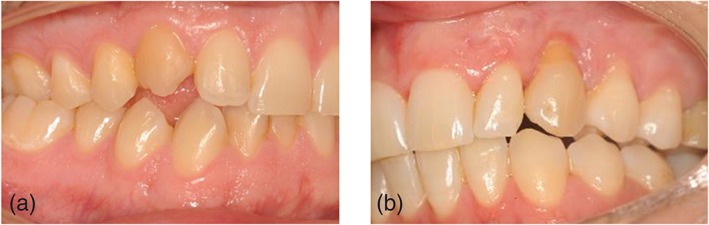
A, Clinical picture of case with ankylosis and infra‐occlusal position of transplanted canine. B, Clinical picture of case with gingiva recession

Almost half of the teeth (13 teeth) had root channel treatment after transplantation. Tooth vitality was examined in the remaining teeth (14 teeth). Five teeth showed a positive result for the cold test. Overall, almost half of the teeth (13 teeth) showed an deepened (>3 mm) clinical pocket depth. Mean pocket depth of the autotransplanted maxillary canines was 3.0 (SD 1.5). Mean pocket depth of the contralateral maxillary canines was 3.0 (SD 1.78). Seven transplanted teeth showed bleeding on probing, meaning moderate inflammation. Six teeth were clinically suspected of ankylosis due to the onset of an open bite (Figure 2,A). On clinical examination major discolouration was seen in 4 teeth. Minor discoloration was seen in 5 teeth. All other transplanted teeth showed normal color.

### 3.2 Aesthetic index

**Figure 3:**
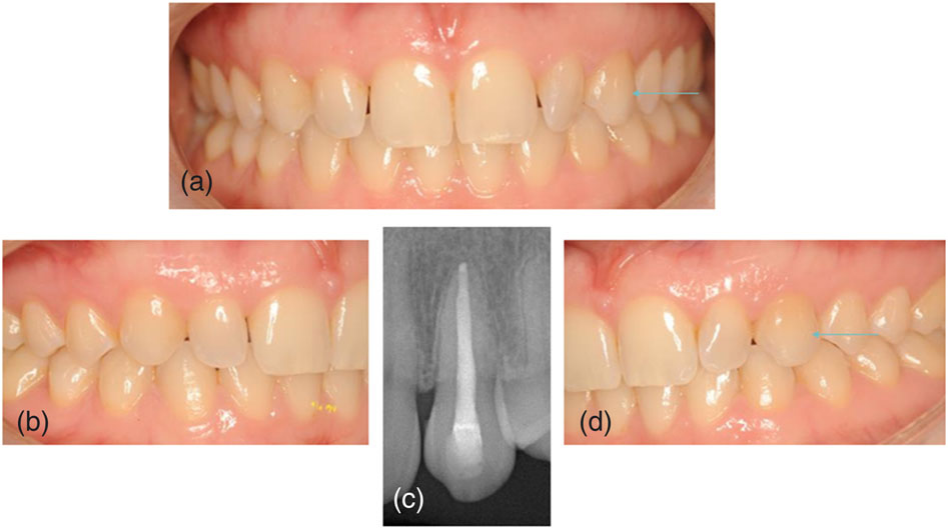
Aesthetic outcome of one case, 18 years after autotransplantation of the left maxillary canine. The tooth had root channel treatment 6 weeks after transplantation. The final functional, aesthetic and radiographic outcomes are excellent

**Figure 4:**
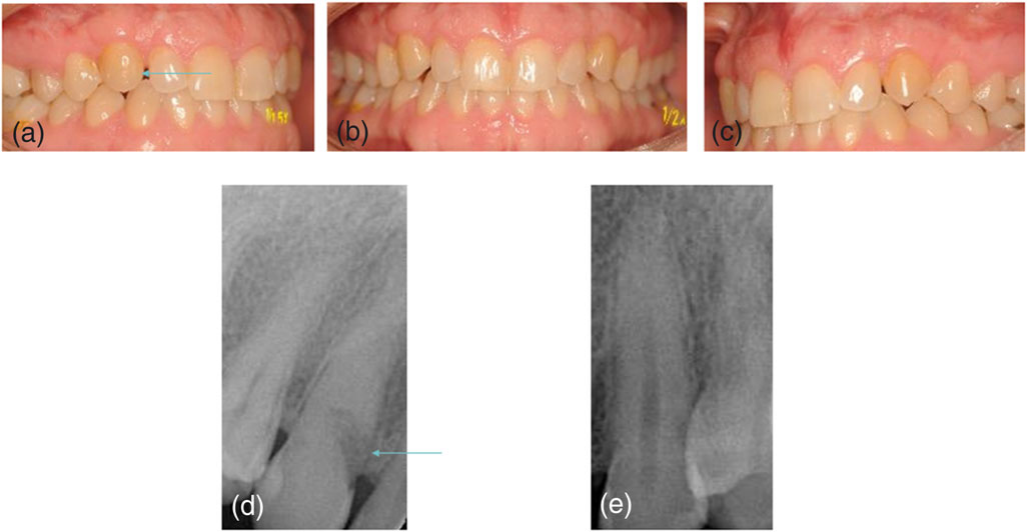
Case of transplanted maxillary canine with sign of resorption. There is an excellent aesthetic outcome with nice gum around the transplanted tooth and good position. However on intra‐oral imaging we can see an external resorption. Also obliteration of the root channel and ankylosis.

Aesthetic outcome of the transplanted canine was assessed with the maxillary canine aesthetic index (MCAI) (Grisar, Claeys, et al., 2018). 16 of the transplanted maxillary canines were scored to have an excellent, 9 with a good, 1 with an acceptable and 1 having a poor aesthetic outcome (Figure 3,4). Two teeth showed extensive recession of the gums (Figure 2,B). Six teeth were found to have a major deviation of the buccolingual inclination when compared to the contralateral maxillary canine.

### 3.3 Radiographical index

Radiographical outcome of the transplanted canine was assessed with the autotransplanted maxillary canine radiographical index (AMCRI) (Grisar, Vanpoecke, et al., 2018). Twelve of the transplanted maxillary canines were scored to have an excellent, 3 with a good, 7 with an acceptable and 4 to have a poor radiological outcome (Figure 2,3,4).

External root resorption was the predominant type of resorption as 9 transplanted teeth showed some sign of external root resorption on 2D and 3D imaging (Figure 4). Three teeth showed apical infection on 2D and 3D imaging. None of the transplanted teeth showed internal root resorption. Four teeth showed signs of ankylosis on 2D and 3D imaging. Three teeth showed apical pathology on 2D and 3D imaging.

### 3.4 Survival rate

**Figure 5:**
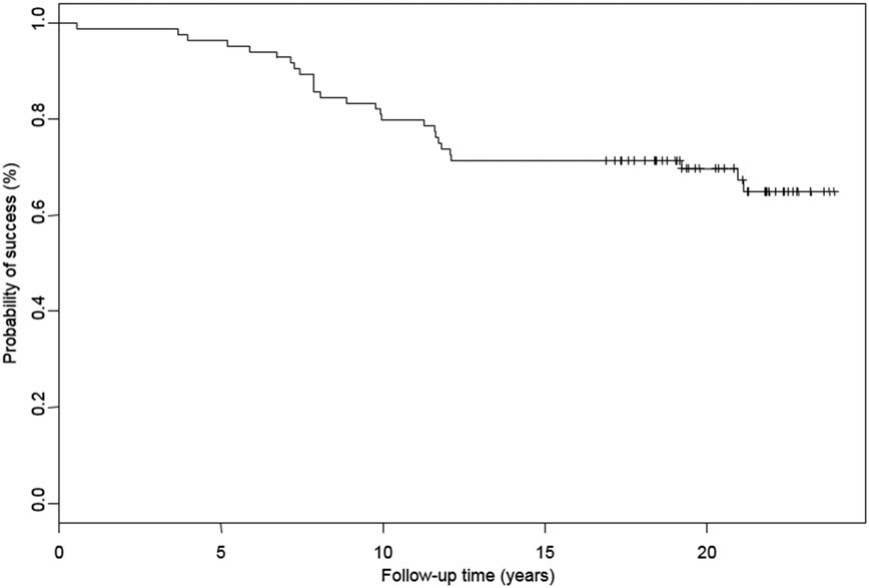
Kaplan–Meier estimation describing the probability of survival for a follow‐up period of 21 years showing a survival rate of 67.9% after 21 years because 27 transplanted teeth were lost before examination

**Figure 6:**
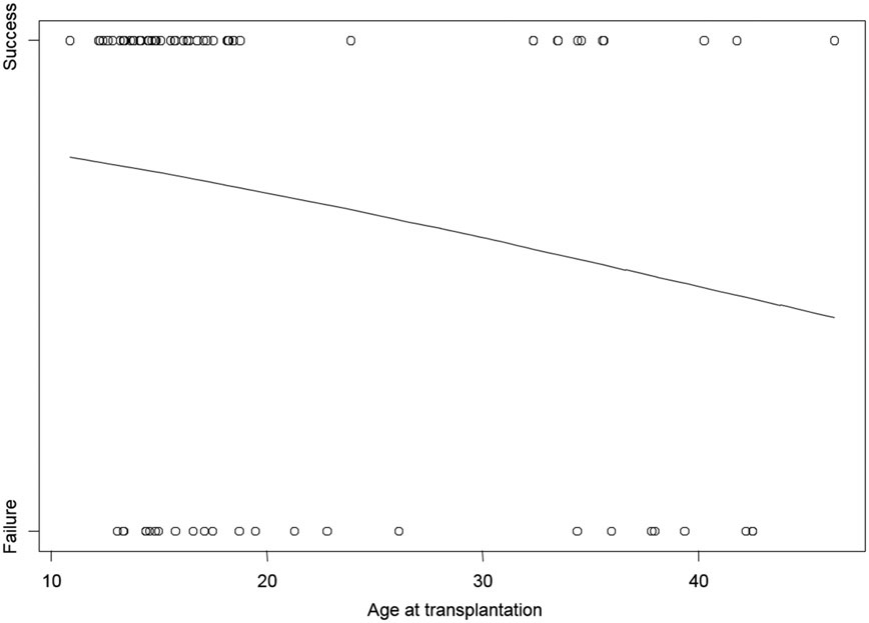
Probability of success as function of age at transplantation. The probability of success decreased when the age at time of transplantation increased (*p* = 0.0966)

Since 27 transplanted teeth were lost prior to endstage examination, the survival rate was 67.9%. Because of a delayed root channel treatment, 1 transplant was lost 6 months after surgery. Figure 5 represents the Kaplan–Meier risk curve for the overall survival rate over 21 years. The mean survival time was 15.8 years (min 0.5 – max 23.9; SD 6.6). Figure 6 shows the relation between age at time of transplantation and survival of the transplanted canine (*p* = 0.0966).

**Figure 7:**
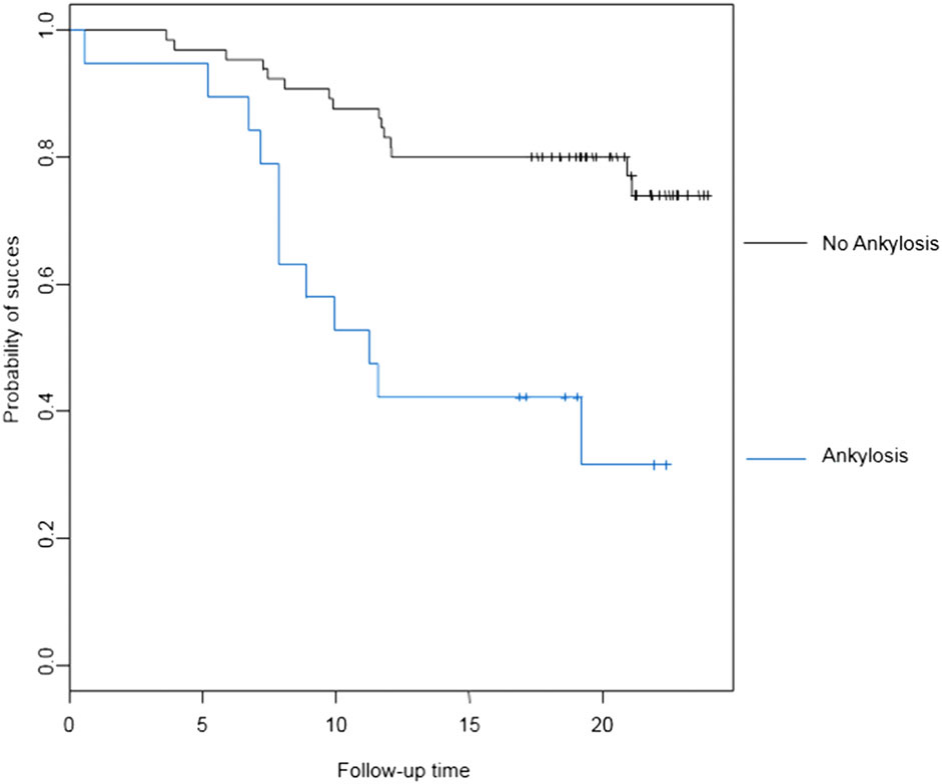
Baseline ankylosis and survival. When pre‐operative investigations uncover ankylosis one should be aware of a higher possibility of failure. Twelve out of 19 ankylosed canines failed after transplantation

**Figure 8:**
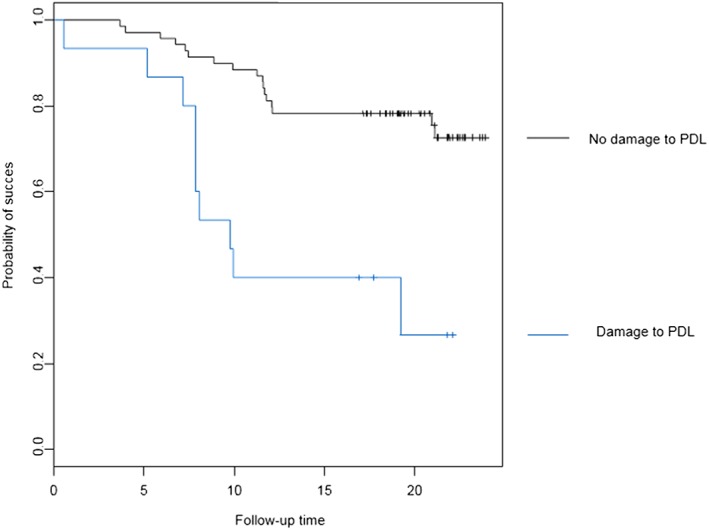
Baseline damage of the PDL and survival

Investigating baseline variables and their influence on final outcome showed a significant correlation between ankylosis of the impacted maxillary canine and failure (*p* < 0.005). Survival analysis correlated with ankylosis as shown in Figure 7. Furthermore, damage to the periodontal ligament during surgical removal of the impacted canine was significantly associated with a worse longterm outcome (p < 0.005) (Figure 8).

### 3.5 Succes rate

The success rate was only calculated for the transplanted teeth that were evaluated on recall visits. In this study was 22 of the 27 surviving transplanted teeth on recall were evaluated as successful after clinical aesthetic and radiological evaluation (Grisar, Claeys, et al., 2018; Grisar, Vanpoecke, et al., 2018).

Patients were questioned using a VAS (visual analogue scale) scoring system consisting of 7 questions:

- Q1: judge retrospectively the overall treatment protocol regarding the inherent therapy and the length of treatment?
- Q2: Does the treatment result fulfill the general expectations?
- Q3: Satisfaction with the treatment outcome from a general aesthetic point of view?
- Q4: Satisfaction with the treatment outcome from a general functional point of view?
- Q5: Satisfaction with the treatment outcome regarding color of the tooth?
- Q6: Satisfaction with the treatment outcome regarding morphology (length and width) of the tooth?
- Q7: Satisfaction with the treatment outcome regarding position of the tooth?

All patients reported high individual scores (average 8.6, range 6.7–9.6), demonstrating a high long term patient satisfaction. Lower VAS scores were related to lower scores on the maxillary canine aesthetic index (MCAI).

### 3.6 Failed transplantations

**Figure 9:**
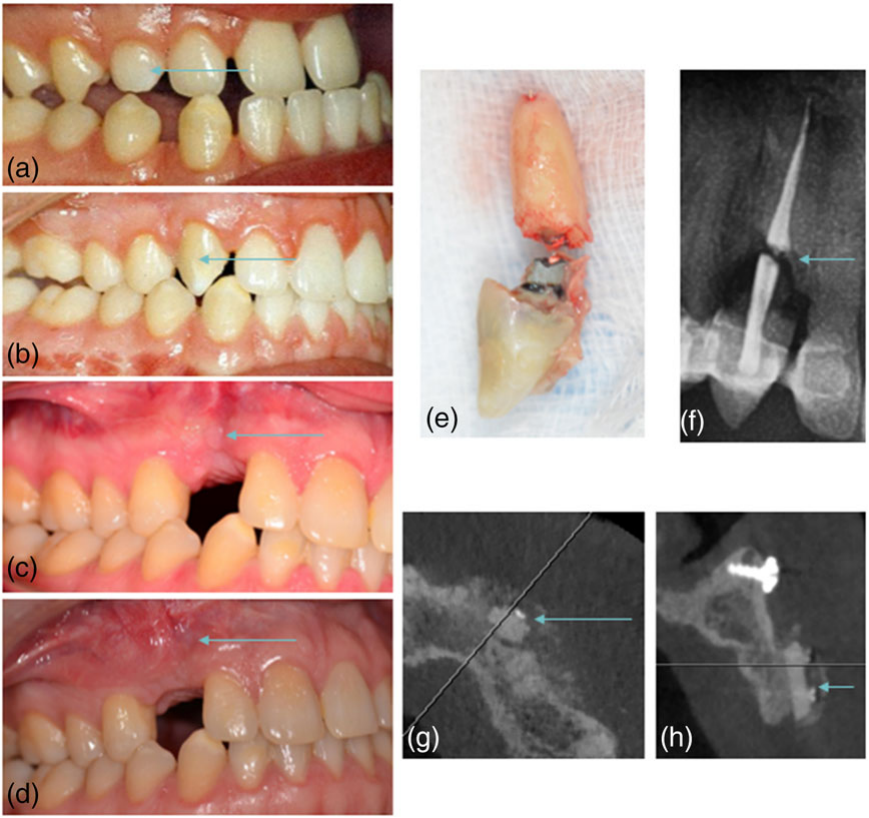
Follow up of a case with failure of the transplanted canine, 19 years after the initial procedure. A, Initial presentation of the patient with primary canine in situ (arrow). B, Clinical outcome 10 years after initial autotransplantation of the impacted right maxillary canine (arrow). C, Clinical image of the gingiva 10 weeks after removal of the failed transplanted tooth with appearance of insufficient bone volume of the aveolar ridge (arrow). E‐F, Clinical and radiographical images of the failed transplanted tooth with clear signs of resorption (arrow). D‐G‐H, Clinical and radiographical images after reconstruction of the alveolar ridge with a ramus bone graft and recovery of vestibular bone volume (arrow)

Further questioning was possible with 18 of the 24 patients with a failed transplanted maxillary canine. Five patients currently have no replacement for the failed transplanted maxillary canine. Seven patients reported succesfull implant replacement while 3 patients had implant surgery planned. In 3 of the 10 cases with (future) implant treatment, a bone augmentation procedure was necessery (Figure 9). Three patients needed replacement with a resin retained bridge.

## 4 DISCUSSION

The survival rate of transplanted maxillary canines in this study, with an average follow up of 21 years, was 67.9%. The mean survival time was 15.8 years. A recent systematic review reported survival ranges to be 88.2% after 5 years or more (Grisar, Chaabouni, et al., 2018). However none of the included studies had a follow up longer then 15 years. A progressive loss of transplanted teeth is to be expected with increasing follow‐up time since it has been proven that with increasing time after transplantation, significantly more root resorption can be expected. (Gonnissen et al., 2010)

When comparing the outcome rate of this study with the literature, it is important to consider the difference in criteria for success, because there are no common success criteria. This study used established criteria for clinical assessment of transplanted teeth. (Altonen, Haavikko, & Malmström, 1978; Patel et al., 2011; Urbanska & Mumford, 1980)

Moreover, the transplanted canine were clinically and radiographically compared with the contralateral canine using previously developed indices. Subsequently, only transplanted canines with an excellent, good or acceptable final aesthetic result, no signs of infection or root resorption and sound periodontal tissues were classified as succesful.

Significant parameters in determining outcome of autotransplantation were baseline ankylosis of the impacted canine and damage of the PDL during surgery as reported by the surgeon.

By questioning the patients with a failed autotransplanted maxillary canine, information was obtained of treatment possibilities after autotransplantation. In current literature, there are no studies investigating the treatment possibilities after loss of autotransplanted maxillary canines. Thus, there is no knowledge of the real complexity of those treatments. In our study population, most patients with failures were enrolled in a non‐complex follow‐up treatment, such as implant surgery without bone grafting or prosthetic replacement. In almost one third of the cases with a dental implant a separate bone augmentation procedure proved to be necessary (Figure 8).

In the present study the authors did not consider transient root resorption, ankylosis or endodontic treatment te be a failure. This because even in case of eventual loss of the tooth, autotransplanted teeth may have been retained for considerable lengths of time, providing an aesthetic and functional solution. However, poor aesthetic or radiological outcomes were considerd to be a failure (Grisar, Claeys, et al., 2018b; Grisar, Vanpoecke, et al., 2018c).

Among the surviving teeth, the longest duration was 23.9 years and the shortest 0.5 years, with an average of 15.8 years. Tooth transplantation is not usually the first line of treatment for patients with impacted canines. (Patel et al., 2011) However, considering a survival percentage of 67.9% after a mean follow up period of 21 years, it should be considered as an option in selected cases.

The use of autogenous transplantation as an alternative for both osseointegrated implants and maryland bridges can be assessed by comparing success rates and survival times for each procedure. The benefits of autotransplantation include the provision of a natural biological tooth and periodontal environment, ensuring a maintenance of the normal exteroceptive function of the tooth to guarantee peripheral feedback and physiological function. (Patel et al., 2011) Just as the potential to induce alveolar bone growth, proprioceptive function, a normal PDL, the potential to erupt with neighboring teeth during continued facial growth while maintaining a normal interdental papilla and allowing orthodontical movement. (Kim, Jung, Cha, Kum, & Lee, 2005; Patel et al., 2011; Zachrisson, Stenvik, & Haanæs, 2004) Moreover, transplantation is possible during growth, in contrast to implant treatments. Viable transplanted teeth have the capacity to further erupt and do not require initial incorporation into bone, when there is sufficient periost surrounding the tooth.

The present study demonstrated an outcome of 67.9% 21 years after transplantation of impacted canines. Baseline ankylosis of the impacted maxillary canine and damage to the periodontal ligament during surgical removal were found to be important prognostic factors, emphasizing the importance of a proper patient selection.

Autotransplantation of impacted maxillary canines may be indicated in selected circumstances, an acceptable long‐term survival rate can be expected. Individual success is difficult to predict and patients must be informed of the potential for failure and associated risks before undergoing such a procedure. (Patel et al., 2011) If this is met, a high patient satisfaction can be expected. If the transplanted tooth is lost, replacement can be achieved by means of a dental implant potentially and in addition requiring a bone augmentation procedure.

## ACKNOWLEDGMENTS AND DISCLOSURE STATEMENTS

The authors report no conflicts of interest related to this study.
